# Reports of Societies

**Published:** 1874-04

**Authors:** 


### Reports of Societies.

***Chicago Society of Physicians and Surgeons.*** *Transactions at Meeting of March* 9, 1874. *Reported by the Secretary.*

The Society met, as usual, in the parlor of the Grand Pacific Hotel, the President in the Chair. The minutes of the preceding meeting were read and approved. Dr. R. H. Bingham, of Castleton Medical College, N. Sk., Class of 1850, was unanimously elected to membership; and the name of Dr. C. T. Parkes, Demonstrator of Anatomy, Rush Medical College, was proposed for consideration of the Censors by Dr. J. E. Owens.

Dr. W. C. Lyman then read an Abstract of Cases treated in the Woman's Hospital of the State of Illinois during the year 1873.* The reading of the abstract was succeeded by some discussion of the treatment pursued.

Dr. P. S. Hayes then read that portion of the Annual Report of the Section on Pathology, having regard to the nervous system. The report was exhaustive of such published material as appeared during the preceding year on the pathology of disorders of nerves and of tissues eminently controlled by the spinal or sympathetic systems. The report was, on motion, accepted.

Dr. John E. Owens, by request of several members, made a verbal report of an operation for disease of the ovary, recently performed by him in St. Luke's Hospital. The report was necessarily imperfect, since the case had not yet been dismissed.

The patient, a female over fifty years of age, and multiparous, presented herself with enlargement of the abdomen to the extent of the distension in pregnancy at the eighth month. The mobility of the mass, subjacent to the abdominal parietes, indicated its non-

* This abstract appears in the pages of the CHICAGO MEDICAL JOURNAL for April, 1874.

attachment to the walls, and the duration of its existence for the previous seven to nine months, seemed to preclude the formation of any firm adhesions to the contiguous viscera. Ether was administered—a small amount of chloroform being added temporarily, as the complete anæsthetic condition was unusually deferred—and an incision carried from just below the umbilicus, to a distance of six or seven inches, exactly in the mesial line. A very few drops of blood were sponged away—as the abdominal walls were greatly attenuated—and the tumor of the ovary exposed. At this point one of its multilocular cysts ruptured, when the patient was at once laid upon her side, and the cystic contents—a substance of the general appearance and tenacity of calves' foot jelly—escaped into a basin—none of it entering the abdominal cavity. The tumor was then readily turned out of its resting place, and its pedicle secured in situ by a clamp. The entire mass was found to consist of a material similar to that already described—presenting the phenomena of colloid degeneration of the ovary in exceedingly typical aspect. A similar degeneration was found to have occurred in the vermiform appendix, the latter having become enlarged to a sextuple dimension, and presenting upon its external aspect minute, cyst-like collections of similar colloid material. The external surface of the small intestine was inflamed to such an extent as to resemble the condition of "granular lids." The lips of the wound were brought together so that the peritoneal surfaces were opposed, and an opening left at the lower extremity into which a pledget of lint was inserted, in order that subsequently, if needful, intra-abdominal injections might be employed. This procedure was, however, found to be unnecessary, as the entire wound has now (10th day) completely closed, and the patient has so far exhibited a very satisfactory condition. The pulse has ranged from 70 to 100 per minute, and the temperature from 98° to 103° F., the exceptional elevation noted having been occasioned by the excitement incident to change of clothing and bedding.

A superficial examination of the morbid material contained in the ovarian cysts, made under the microscope by Dr. I. N. Danforth, revealed the presence of trabeculæ, described by Paget as "fibrous white cords, or thin membranes, arranged as in the areolar tissue, or in a wide-meshed net-work."

The narration of the details of this case elicited a brief discussion.

The speaker (Dr. Owens) then read the following abstract of cases occurring in St. Luke's Hospital:

I. *Parietal Gaseous Abscess of the Abdomen.*

The formation of abscess in the walls of the abdomen is of unfrequent occurrence. The matter may be superficial, it may collect between the layers of muscles, or lastly, between the latter structures and the peritoneum. Some are connected with disease within the cavity; others again are uncomplicated. When deep-seated the abscess is said to imbibe gas from the intestinal tube, which is very apt, as the disease advances, to become adherent to the posterior wall of the abscess.* When deep-seated, muscles and aponeuroses bind down the pus, and it is consequently a long time in coming to the surface.

September 17th, 1873, an unmarried girl, aged nineteen years, was admitted to St. Luke's Hospital, with vaginitis. The case was complicated by a somewhat diffused inflammatory induration and swelling situated in the lower part of the abdomen on the left side. This swelling began about a month previous to the patient's admission to the hospital. By means of pressure by the bandage these signs of inflammation had so far subsided that the patient was discharged Oct. 6. The animal functions were apparently normal.

December 2nd, probably at least three and a half months from the beginning of the inflammation, and two and a half months after her discharge, the patient was re-admitted to the hospital. She had been at work; the inflammation had grown more diffuse, swelling, induration and pain were prominent symptoms, and inflammatory fever had already supervened.

The patient having been put to bed, fomentations were applied to the part, and morphia administered in sufficient quantity to allay pain. The inflammation was limited below by Poupart's ligament; internally, by the linea-alba; superiorly, by a transverse line passing through the umbilicus; externally, by a line touching the anterior-superior-spinous process.

Four or five days subsequent to re-admission, a soft spot was discovered, in which one could place the end of the fore finger. That the little cavity contained pus was pretty certain. It possessed also an unusual element in its contents, viz., a bubble of gas. The following day the cavity had enlarged, and the quantity of pus and gas had proportionately increased. Upon manipulation the bubbling was both felt and heard. Percussion yielded, not the usual flat sound of an abscess, but the tympanitic sound of gas accumulation. So far as operative interference was concerned, nature was left to take her course, and this plan is a good one, especially, if the pus shows a disposition to come to the surface. Gradually the cavity enlarged; pus and gas became more abundant, and in ten days from the date of re-admission the cuticle sloughed, giving exit to a large quantity of offensive sanious pus, and very offensive gas (sulphuretted hydrogen). Convalescence was rapid, and the patient was discharged December 23rd.

* Gross.

II. *Empyema—Paracentesis—Great Improvement from the use of Drainage Tube.*

M. O. Heydock, M.D., in charge of the medical wards of the hospital, and in charge of this patient, has kindly furnished me the following notes in the case:

"Charles Sparks, aged twenty-one years, was admitted into St. Luke's Hospital, February 22nd, 1872, and gave the history of a recent attack of pleuro-pneumonia. The characteristic signs attendant upon the presence of fluid in the pleural cavity were easily recognized. He was subjected to the treatment ordinarily resorted to, under like conditions, until May 17th. At this time the vital powers were rapidly failing. Hectic, emaciation, diarrhœa, and oppressed breathing, foreshadowed a fatal issue, and as a last resort, paracentesis was performed by Dr. Owens, giving exit to five pints of pus. On the 23rd of the same month a second operation was performed, giving exit to two pints of pus. A drainage tube was introduced, and the cavity daily washed out with a weak solution of carbolic acid in water. The discharge gradually diminished in quantity, and there was in all respects a remarkable change for the better in the condition of the patient. Under the influence of good diet and tonics, as iron, quinine and cod liver oil, June 8th found him walking about the wards of the hospital, and July 20th out of doors, the discharge at this time being some two ounces of pus per day.

"July 30th it was thought best to remove the drainage tube, lest its presence might be operating as a seaton, and in a degree, at least, furnish an explanation why this amount escaped daily. The upper orifice rapidly closed, and the discharge from the lower grew small in quantity. During the month of August he convalesced rapidly. Early in September the discharge grew thin and offensive, the patient lost his appetite, diarrhœa and night sweats set in, and rapid emaciation followed. There was tenderness in the axillary region, bulging and redness of the upper cicatrix, and the condition of the patient more unpromising than at the time of the first operation.

"Recourse was had once more to the knife, exit given to a large quantity of unhealthy pus, and the tube re-introduced September 18th, 1872. Little, if any, constitutional disturbance followed a somewhat tedious operation, during which the patient was under the full influence of an anæsthetic. Hectic and diarrhœa rapidly disappeared, the appetite returned, and soon after the patient was sent home, and from that time until the present—nearly one and a half years—he has worn the drainage tube, and enjoyed, during a very considerable portion of the time, a fair degree of health and strength."

A few days ago the patient was admitted to the surgical wards.

The old tube is growing weak, from having been long bathed in pus, and the patient desires a new one, if necessary. Can the tube be altogether dispensed with? The respiratory murmur can be heard more or less distinctly over the whole extent of the lung posteriorly. The murmur is distinct over the upper part of the chest, but it cannot be detected in ordinary respiration over the lower third of the lung in front, but it can be when forced breathing is employed. Six or eight weeks ago an exfoliation of bone about an inch long was removed by the patient from the anterior orifice. The posterior opening is in the intercostal space of the seventh and eighth ribs, on a line dropped from the posterior border of the axilla. The anterior is located between the third and fourth ribs, immediately above the nipple. The discharge at present does not exceed two ounces daily. The orifice is well washed with a weak solution of carbolic acid.

III. *Amputation According to the Method of Esmarch.*

The stump, left after an amputation of a leg which had been crushed by an injury, exposed the ends of both bones at the bottom of an indolent ulcer, which completely precluded the application of an artificial limb. Re-amputation was performed, after the bloodless method, by encircling the limb with a rubber bandage, each successive turn of which overlapped that which preceded. The elastic tubing was applied immediately upon the last turn of the roller. The leg had been previously suspended, in order to obviate the possibility of the formation of a clot in the vessels, which might induce a thrombosis and subsequent embolism elsewhere. The operation was absolutely bloodless—as much so as in the dissection of a cadaver. Subsequently a very small slough occurred upon the edge of the flaps in the site of an old cicatrix, and where the tissues were superimposed upon a layer of cartilage, no greater, however, than is frequently seen under the most favorable circumstances. The operation, in its issue, was eminently successful.

The discussion of these cases turned mainly upon the question of the propriety of opening abscesses of the abdominal parietes with gaso-purulent contents.

Dr. Jackson asked whether it were well to neglect the golden rule of surgery, to give exit to pus which was obviously seeking an external outlet.

Dr. Merriman asked if there was not danger of such an abscess bursting internally, its contents finding their way into the abdominal cavity.

Dr. Owens thought it well to consider the possibility of the formation of an artificial anus as a sequel to such interference.

Dr. Lyman thought that surgical records might be searched in vain for an instance of such an abscess finding an exit to the peritoneal cavity, since the process of agglutination of its lining membrane, which proceeds in advance of the approaching abscess, attaches to the latter a coil of adjacent intestine, into which the contents of the tumor necessarily escape.

Dr. Hyde thought that the presence of gas in an abdominal abscess, implied the proximity of an adherent coil of intestine. In purulent and fetid collections in the neighborhood of the rectum, there is generally some rent in the rectal wall, establishing a communication between the cavity of the abscess and the gut; and in cases of abscess of the walls of the abdominal parietes where no gut adhesion has occurred, as in the instances caused by urinary infiltration upward from a false urethral passage, there is ammoniacal fetor and sloughing, but no gas.

Dr. Wickersham considered that the spontaneous opening of the abscess, when it evidently pointed externally, should be confidently and patiently awaited. He narrated a case, occurring in his own practice, in which a boy fell upon his abdomen while at play, and in two or three days subsequently suffered from dysuria. It was doubtful whether the excruciating pain which he endured, resulted from lesion of the bladder, peritoneum or bowels. So severe was the latter symptom that one-third of a grain of morphia was requisite to procure relief for the little patient, who was but a few years old. The catheter was used twice daily, and was necessitated by the presence of an indurated mass, which soon became detectable, situated, apparently, in a plane posterior to the bladder. In three or four days afterward, fluctuation became evident, and a spontaneous exit of pus occurred at the navel, in quantity equal to three or four tablespoonsful. The pain soon subsided, and the little patient made a good recovery.

Dr. Merriman thought that occasionally there was danger of a spontaneous opening, *both* externally and internally, and cited the case of a lady, then under his professional care. There had been, several years ago, a pelvic abscess which had resulted in such a manner that now, as indeed during the entire period since the occurrence, there were alternate intervals of a few days, in which there were feculent discharges from the bladder and urinous discharges from the rectum.

The Secretary stated, that in his belief a majority of such cases had a history of first, spontaneous bursting of the abscess in one direction, and subsequently, ulceration and irritation from the presence of foreign secretions, sufficient to result in the formation of a counter-opening. He alluded to the testimony of pathological anatomy as bearing upon the question at issue.

The discussion was then closed.

It was resolved, on motion of Dr. Owens, that every member of the Society connected with the staff of a hospital, be added to the "Committee on Clinical Reports."

It was announced, that at an early date, Dr. J. H. Etheridge would read a paper on the "Organic Hydrides," and Dr. John Bartlett would present the "Annual Report of the Section on Pathology."

The Secretary extended an invitation to the members of the Society, from the Chicago College of Pharmacy, to attend the Annual Exercises of the College on the evening of the 10th inst.

The Society then adjourned.

***Central Illinois Medical Society.***

The Central Illinois Medical Society met in semi-annual session at Clinton, Tuesday, Dec. 9, 1873. Called to order by Dr. W. Hill, of Bloomington, President. Opened with prayer by Rev. Mr. Piper, of Clinton.

Dr. J. Wright, Chairman Committee of Arrangements, in a brief address, bid the Society a thrice hearty welcome to Clinton and Clinton hospitalities; to which Dr. Laughlin, of Bloomington, on the part of the Society, briefly responded.

Minutes of previous meeting read and approved.

T. W. Davis, M.D., of Wapella, D. W. Edmiston, M.D., B. S. Lewis, M.D., of Clinton, J. H. Tyler, M.D., A. L. Norris, M.D., of DeWitt, Elias Wenger, M.D., of Gilman, C. R. Carr, M.D., of Bloomington, J. M. Waters, M.D., of Gibson, were recommended for membership, and Censors reporting favorably, they were duly elected.

Drs. M. G. Parsons, of Jackson county, Hunt, Adams, and Mahern, of Clinton, Axton and Crothers, of Maroa, being present, were invited to participate in the proceedings of the Society.

On motion, adjourned till 2 P. M.

AFTERNOON SESSION.

Called to order by President Hill. Minutes of morning session read and approved.

Dr. Jno. Wright, of Clinton, read an able report on "Puerperal Convulsions." He did not think uræmic poison a cause. He thought blood-letting advisable in some cases; others, not; that different cases require different treatment.

Dr. Pearman, of Champaign, thought sulphate of atropia a good agent to control convulsions. Dr. Miller, of Farmer City, reported a case in which hydrate of chloral, bromid of potassium, and sulph. morph. exercised a very favorable influence over convulsions. Did not think he derived benefit from blood-letting. Dr. Goodbrake, of Clinton, had never seen any benefit derived from blood-letting. His experience warranted the use of chloroform, and, thinking uræmic poison often an exciting cause, would use diuretics. Dr. Parsons, of Jackson county, thought he had derived great benefit from fluid extract of scutellaria. Dr. Birney, of Urbana, thought uræmic poison an exciting cause.

Dr. S. H. Birney, of Urbana, Chairman of Committee on Surgery, read an interesting report on "Surgery: its Social and Legal Status," in which he spoke at length of malpractice. He thought suits to recover damages were often brought against surgeons by the intervention of meddlesome physicians (so called.) He was much opposed to men not medical sitting as jurymen in suits for malpractice, and thought the laws of the State of Illinois very unjust in requiring so much of surgeons, without allowing them the necessary privileges for obtaining the knowledge necessary to become good surgeons, viz: that of dissecting.

Dr. J. Little, of LeRoy, of Committee on Surgery, read an able report on "Amputation at the Knee Joint." He had performed this operation with the most satisfactory results, and thinks it safer than amputation at the upper joint of the leg, and leaves a better stump for an artificial limb.

Dr. S. H. Birney, Chairman of Committee on Surgery, read an excellent report on "Shock." He had great confidence in the restorative properties of nature. Would not give alcoholic stimulants; if any stimulant, would give ammonia. This report elicited a lively discussion. Drs. Hill, of Bloomington, and Goodbrake, of Clinton, would resort to alcoholic stimulants.

EVENING SESSION.

Called to order at 7 o'clock P. M. by President Hill.

Dr. J. T. Pearman, of Champaign, read a very interesting report on "Practical Medicine," in which he spoke at length of typhoid fever, its cause and treatment. He thinks it is an enteritis, and that turpentine is the sheet anchor in its treatment; that turpentine is as much a specific for typhoid fever as quinine is for intermittent fever. Dr. Goodbrake, of Clinton, thought it was not a self-limited disease; that it need not necessarily run a certain number of weeks.

The hour having arrived for the "Public Address," Dr. T. F. Worrell, of Bloomington (President Illinois State Medical Society), delivered an ably prepared address to a house full of Clintonians, in which he spoke at length of the obligations of physicians to patients, and patients to physicians.

On motion of Dr. C. Goodbrake, the thanks of the Society were respectfully tendered Dr. Worrell, for his able address, and he was requested to furnish a copy of it for publication in the CHICAGO MEDICAL JOURNAL.

Dr. R. G. Laughlin, of Bloomington, Chairman of Committee on Practical Medicine, read a practical and very interesting report on the different diseases met with since the last meeting of the Society, in which he spoke of cholera, in treatment of which he suggested the use of milk by injection. He also spoke of cholera infantum, yellow fever, typhoid fever, etc. Dr. Worrell advocated very strongly the use of verat. viride in treatment of typhoid fever. Dr. Goodbrake thought brandy a better agent for reducing frequency of the pulse.

Dr. C. T. Orner, of Saybrook, read a brief and interesting report on "Obstetrics," in which he made some valuable suggestions to the physician in the lying-in room. He also suggested that in cases of foot and breech presentations, where the body is delivered and head remains undelivered, that "instead of putting the index finger in the mouth, and rounding the body up over the os pubis, that the *left* hand be placed under body of child, and supported on a line nearly level with the body of the woman, while with the right hand, between pains, the child is pressed back against the womb, till the frontal portion of the head strikes the sacral angle; thus producing extreme flexion of the head, and disengaging it from the os pubis." The advantages claimed being the presentation of the short diameters of head, the increased power of the uterus, the prevention of pressure on the cord, and birth of child at first pain. The Doctor supported this method by reporting cases in which he had practiced it with the most satisfactory results; also the experience of his friend, Dr. J. B. Brooks, of Reading, Pa., who had practiced it in a number of cases, and to whom Dr. Orner was under obligations for the above suggestions.

Dr. W. G. Cochran reported a case of monstrosity, in which a child of six months' development was born, with evidence of having been dead some time, with a fleshy tumor in front of the neck, as large as the head of a fully developed fœtus, and, in connection with the placenta, a semi-fleshy mass, which, with placenta, weighed six pounds.

Delegates to American Medical Association: Drs. Elias Wenger, R. G. Laughlin, W. L. Pollock, S. H. Birney, Jno. Wright.

Delegates to State Medical Society: Drs. W. Hill, W. G. Cochran, T. D. Fisher, J. T. Pearman, C. Goodbrake, H. C. Howard.

SPECIAL COMMITTEES.

*Surgery.*—Drs. H. C. Howard, C. Goodbrake, J. M. Waters.

*Practical Medicine.*—Drs. T. D. Fisher, W. L. Pollock, E. C. Bartholow.

*Obstetrics and Diseases of Women.*—Drs. E. Wenger, J. S. Miller, J. L. White.

*Microscopy.*—Dr. R. H. Huddleston.

*Diseases of Eye and Ear.*—Drs. C. R. Carr, D. W. Edmiston.

*Hypodermic Medication.*—Dr. Jno. Wright.

*Skin Grafting.*—Dr. J. T. Pearman.

*New Remedies*—Dr. J. Little.

*Progress of Physiology.*—Dr. A. L. Norris.

Next place of meeting: Bloomington.

Committee of Arrangements: Drs. T. F. Worrell, R. G. Laughlin, J. L. White, H. C. Luce.

On motion, the Society adjourned at midnight, to meet in Bloomington, the second Tuesday in June, 1874.

W. G. COCHRAN, *Sec'y*

